# Summer Buffalo Milk Produced in China: A Desirable Diet Enriched in Polyunsaturated Fatty Acids and Amino Acids

**DOI:** 10.3390/foods11213475

**Published:** 2022-11-02

**Authors:** Pei Nie, Bin Pan, Muhammd Jamil Ahmad, Xinxin Zhang, Chao Chen, Zhiqiu Yao, Haimiao Lv, Ke Wei, Liguo Yang

**Affiliations:** 1 College of Veterinary Medicine, Hunan Agricultural University, Changsha 410128, China; 2 Key Laboratory of Animal Genetics, Breeding and Reproduction, Ministry of Education, College of Animal Science and Technology, Huazhong Agricultural University, Wuhan 430070, China; 3 International Joint Research Centre for Animal Genetics, Breeding and Reproduction (IJRCAGBR), Huazhong Agricultural University, Wuhan 430070, China; 4 Hubei Province’s Engineering Research Center in Buffalo Breeding and Products, Wuhan 430070, China

**Keywords:** fatty acids, amino acids, milk, crossbred buffalo, summer, winter

## Abstract

The objective of the study was to compare and reveal differences in basic chemical parameters, fatty acids, amino acids, and lipid quality indices of crossbred buffalo (swamp x river type) milk produced in summer and winter. The buffalo milk samples were collected in summer (Jul–Aug) and winter (Dec–Jan) from Hubei province, China. The samples were detected by using CombiFoss apparatus, gas chromatography, and an automated specialized amino acid analyzer. The results showed that the basic chemical parameters, fatty acid profiles, lipid quality indices, and amino acid profiles of crossbred buffalo milk differed between summer and winter. Specifically, summer buffalo milk exhibited a higher content of MUFA (monounsaturated fatty acids) and PUFA (polyunsaturated fatty acids) than winter buffalo milk. Summer buffalo milk had a lower content of major SFA (saturated fatty acids), a higher content of ω-3 and DFA (hypocholesterolemic fatty acids), a lower ω-6/ω-3 ratio, a higher value of 3 unsaturated fatty acid indices (C14, C16, C18), and a lower value of IA (index of atherogenicity) and IT (index of thrombogenicity) than winter buffalo milk. Additionally, 17 amino acids, including 8 EAA (essential amino acids) and 9 NEAA (non-essential amino acids) were higher in summer buffalo milk. These results indicated that summer buffalo milk was more health-beneficial than winter buffalo milk. Therefore, summer buffalo milk might be a desirable diet option for human nutrition and health. Our findings provide valuable information for the research and development of buffalo dairy products in China or other Asian countries.

## 1. Introduction

According to data reported to FAO, the buffalo (*Bubalus bubalis*) is the second largest source of milk worldwide, exhibiting an increasing milk production capacity, reaching 134 billion liters in 2020, accounting for 15% of total milk production. Buffalo milk is a nutrient-rich daily sustenance and plays an important role in maintaining human nutrition and health. It is mainly produced in some developing countries with over 99% of buffalo milk produced in India, Pakistan, China, Egypt, and Nepal [1]. Just the milk of cows, goats, sheep, donkeys, camels, and mares, buffalo milk also contains multiple nutrients such as fat, protein, polyunsaturated fatty acid, and essential amino acid, and buffalo milk enriches options of dairy products (Table 1) [1,2,3,4].

Hence, buffalo milk and its dairy products have become increasingly popular in some Asian and European countries in recent decades. In some Asian countries such as India, Pakistan, Egypt, and Nepal, the consumed fresh buffalo milk accounts for over a half of liquid milk. Moreover, other buffalo dairy products, including butter oil, dahi, and yogurt, as the traditional foods, are widely consumed by Asian [11]. In Europe, some Mediterranean countries, especially Italy and Turkey, buffalo milk is mainly used to produce various dairy products, such as liquid milk, butter, ghee, cheeses, ice cream, yogurt, and buttermilk. Their buffalo industry is booming and has gained high popularity. Mozzarella cheese produced from buffalo milk is of a high market value, and it is also under the European Union’s protected designation of origin scheme [12,13]. Generally, the buffalo milk price is threefold times as much as that of cow milk [14]. In addition, the nutritional advantages of buffalo milk make it a potential substitute for cow milk, especially for those consumers with milk allergies [11,12,15], which will expand the market of buffalo milk industry and promote its prosperity. 

Over the past thirty decades, numerous researchers persistently paid attention to the milk fatty acids (FA), including more than 400 individual fatty acids, most of which contain natural fat and variable macronutrients [16,17,18,19]. Fatty acids can be divided into short-chain fatty acids (SCFA), medium-chain fatty acids (MCFA), and long-chain fatty acids (LCFA) based on the number of carbon atoms (C = 4–11, C = 12–16, and C ≥ 17) (LCFA). Based on the number of double bonds between carbon atoms, FA are usually categorized into three groups, namely, saturated fatty acids (SFA), monounsaturated fatty acids (MUFA), and polyunsaturated fatty acids (PUFA). SFA are further classified into straight-chain fatty acids and branched-chain saturated fatty acids (BCSFA), and latter includes odd-chain saturated fatty acids (OCSFA) and even-chain saturated fatty acids (ECSFA). Moreover, FA are also classified into hypocholesterolemic fatty acids (DFA) and hypercholesterolaemic fatty acids (OFA) in terms of its relevance to human diseases [16,20,21]. Generally, milk fatty acids have been confirmed to have beneficial and passive influence on human nutrition and health. For example, SCFA and MCFA have been reported to have the positive effects on obesity, and odd brain-chain fatty acids (OBCFA) exhibit beneficial effects on inflammation. However, some adverse effects of LCFA and MCFA (14:0; 16:0, and 18:0) on cardiovascular disease and inflammation have been found in some studies [22,23,24,25]. PUFA contain two essential fatty acids, linoleic acid (LA, 18:2; ω-6) and alpha linolenic acid (ALA, 18:3; ω-3), which cannot be synthesized by human autologously but can obtained from milk [26]. Some studies have revealed that diets rich in ω-6 might accelerate inflammation, thrombosis, arteriosclerosis, and coronary heart disease, and other non-infectious diseases [27,28]. Conversely, ω-3 has a positive impact on arrhythmia, neurological development, and immunomodulation [29,30]. During the last hundreds of years, the ω-6/ω-3 ratio of the Western diet has been up to 20:1, which is far above the ratio of 5:1 to 10:1 recommended by the WHO [31]. Milk is an essential source of dietary lipids, and fatty acids in milk have received increasing attention. Most existing studies have focused on cow milk, and relatively fewer studies have been conducted on buffalo milk [20,32,33,34,35,36,37,38,39,40,41].

As an important dietary protein source, milk enjoys great popularity in the world. Milk protein, which is rich in numerous essential amino acids, arouses researchers’ great interests due to its high biological value, good digestibility, rapid absorption, and utilization [42]. Only a small amount of essential amino acids (EAA) is synthesized by the body, or even some EAA cannot be synthesized at all, which cannot satisfy the human’s needs for nutrients. Milk is rich in eight EAA, including histidine, isoleucine, leucine, lysine, methionine, phenylalanine, threonine, and valine, and thus milk with 8 EAA is consumed for multiple purposes such as daily nutrition, sport and exercise supplement, infant formulas, and dietary therapeutical formulas [43,44,45,46]. Likewise, branched chain amino acids (BCAA) consisting of isoleucine, leucine, and valine (accounting for ~40% of EAA in milk) play an important role in protein metabolism, and as nitrogenous precursors, BCAA are used to synthesize glutamate, glutamine, alanine, and aspartate in the mammary gland [47]. BCAA can regulate physiological and metabolic processes. BCAA are related to breast health and milk quality, and they are involved in lipolysis, glucose metabolism, glucose transit, intestinal barrier function and absorption, embryo development, and immunology [48,49,50,51].

Due to its abundant nutrients, buffalo milk can meet certain human’s nutritional needs, and the buffalo dairy industry exhibit economic potentials. However, there is little research on crossbred buffalo milk in China. Therefore, this study aimed to reveal difference in chemical parameters (fat, protein, lactose, and total solid), fatty acid profile, and amino acid profile between summer buffalo milk and winter buffalo milk produced in China. 

## 2. Materials and Methods

### 2.1. Animals

Twenty-four crossbred buffaloes were selected from the farm of Jinniu Animal Husbandry Co. Ltd., Wuhan, China. A clinical examination and hematological evaluation were conducted to ensure that the experimental animals were healthy. The breeding conditions were sanitary and dry. All the animals were fed with a total mixed ration consisted of corn silage, peanut vine, rice straw, and mineral brick, and under the stall-feeding system and with free access to the fresh and clean water.

### 2.2. Sampling

The crossbred buffalo milks were divided into two groups summer milk and winter milk. The summer milk was collected in August 2013 each week as a batch, with 4 batches (12 samples) in the summer milk group and 3 milk samples per batch. The winter milk was collected from the same herd in January 2014 each week as a batch with 4 batches (12 samples) in winter milk group and 3 milk samples per batch. A sample consisted of two milk samples. The first milk sample (200 mL) was collected at 6:00 and refrigerated at 4 °C. The second milk sample (100 mL) was collected at 18:00. The second milk sample was pooled with the first, and then 250 mL of the mixed sample was immediately frozen and transferred to −20 °C storage until analysis for fatty acids and amino acids. The remaining was stored at 4 °C for milk parameter detection. All the experiments were performed in three replicates.

### 2.3. Analysis Methods

#### 2.3.1. Milk Chemical Parameters

The collected milk samples were sent to the Hubei Dairy Herd Improvement Technology within 24 h for basic milk parameters (fat, protein, lactose, total solids) by CombiFoss FT + (Foss, Hillerød, Denmark).

#### 2.3.2. Fatty Acids Profiles

According to AOAC, first, lipids were extracted; subsequently, fatty acid methyl esters (FAMEs) were extracted with hexane. The methyl esters were analyzed by the gas chromatography (GC) method [52]. The obtained fatty acid profiles were analyzed using a gas chromatography (7890A, Agilent Technologies, Santa Clara, CA USA) equipped with a flame ionization detector (FID) and an HP-FFAP capillary column (DB-23, 30 m × 250 μm × 0.25 μm). The inlet temperature was set as 230 °C, which was steadily increased to 230 °C at 3 °C/min. The FID temperature was set as 280 °C. High-purity nitrogen was used as carrier gas, and injection volume was 1 μL under at a split ratio of 1:10.

FA composition was expressed as grams per 100 g in total fatty acids. A total of 37 fatty acids were detected. Only the fatty acids accounting for more than 0.02% of total fatty acids were reported. All FA measures were accomplished by the Chinese academy of agricultural science (Oil Crops Research Institute) in Hubei.

#### 2.3.3. Amino Acid Profiles

According to the AOAC, the amino acid profiles of buffalo milk were analyzed with an automated specialized amino acid analyzer (L-8900, Hitachi, Tokyo, Japan) [53]. A thawed milk sample (10 mL) was completely mixed and hydrolyzed using isometric HCl (10 mL, 6 mol/L) in a sealed hydrolysis tube firstly. Three or four drops of phenol was added to the hydrolysis tube and then frozen for 3–5 min. Subsequently, the acid hydrolysis tube was filled with nitrogen, and samples were hydrolyzed for 22 h at 110 °C. The solution was diluted with 200 mL of deionized water and filtered by a filter (0.45 μm), vacuum dried and dissolved with 1 mL buffer (pH = 2.2). The analysis of AA was performed by using high performance anion exchange chromatography with pulsed electrochemical detection (HPAEC-PED) [54]. The results were expressed as a percentage of total amino acids. All AA measures were conducted by the Chinese academy of agricultural science (Oil Crops Research Institute) in Hubei.

Lipid quality indices of buffalo milk included saturated (C14) index (SI14), saturated (C16) index (SI16), saturated (C18) index (SI18), atherogenicity index (AI), thrombogenicity index (TI), hypocholesterolaemic/hypercholesterolaemic ratio (HH), PUFA ω-6/PUFA ω-3 ratio(ω-6/ω-3). The following formulae were used to calculate lipid quality indices based on fatty acid composition:

Indices of Unsaturated fatty acid [36] 

C14 Index =C14:1/(C14:0 + C14:1)

C16 Index =C16:1/(C16:0 + C16:1)

C18 Index = C18:1/(C18:0 + C18:1)

Index of Atherogenicity (IA) [55]

IA= (C12:0 + (4 × C14:0) + C16:0)/(ω-3 + ω-6 + MUFA) 

Index of Thrombogenicity (IT) [55]

IT= (C14:0 + C16:0 + C18:0)/(0.5 × C18:1 + 0.5 × other MUFA + 0.5 × ω-6) + (3 × ω-3 + ω-3/ω-6)

Hypocholesterolemic/hypercholesterolemic ratio (HH) [56]

H/H = (C18:1+ PUFA)/(C12:0 + C14:0 + C16:0)

PUFA ω-6/PUFA ω-3 ratio(ω-6/ω-3) [57]

ω-6/ω-3= ∑PUFA ω-6/∑PUFA ω-3

PUFA/SFA (P/S) [57]

P/S= (∑PUFA ω-6 + ∑PUFA ω-3)/(C4:0 + C6:0 + C8:0 + C10:0 + C12:0 + C14:0 + C15:0 + C16:0 + C17:0 + C18:0 + C20:0

### 2.4. Statistical Analysis

One-way analysis of variance (ANOVA) with the Duncan’s test was used to investigate the significant difference in chemical parameters, individual fatty acids, fatty acids groups, lipid quality indices, individual amino acids, and amino acids groups between the summer group and the winter group. *p* < 0.05 was considered as statistically significant. 

## 3. Results and Discussion

### 3.1. Chemical Parameters of Crossbred Buffalo Milk

As shown in Table 2, there were some differences in several chemical parameters between summer and winter buffalo milk. Compared with summer milk, winter milk contained more fat, lactose, and total solids, but less protein. Our results could be explained by one previous report that chemical parameters’ differences in milk quality are related to a summer diet with more fresh grass and a winter diet with more mixed silage in six Holstein Friesian dairy cows [58]. However, it is worth noting that winter milk had a significantly higher (*p* < 0.05) lactose content, which is opposite to a previous report on buffalo milk of Italy [59]. The different results in two studies might be associated with somatic cells, which is negatively related to lactose content. Somatic cells in milk are thought to be a sign of buffalo mastitis directly affecting milk lactose content. Low level lactose indicates the diminished secretory cell activity in mammary gland tissue, suggesting the presence of mastitis, which was supposed by another study result that showed that the lactose content in milk produced by buffaloes with mastitis dropped in 2010 and 2020 [59,60,61,62]. Another study on 72 farms in 2015–2020 reported milk lactose as a biomarker of subclinical mastitis in dairy cows [63].

### 3.2. Fatty Acid Profiles and Lipid Quality Indices in Crossbred Buffalo Milk Fat

**Figure 1:**
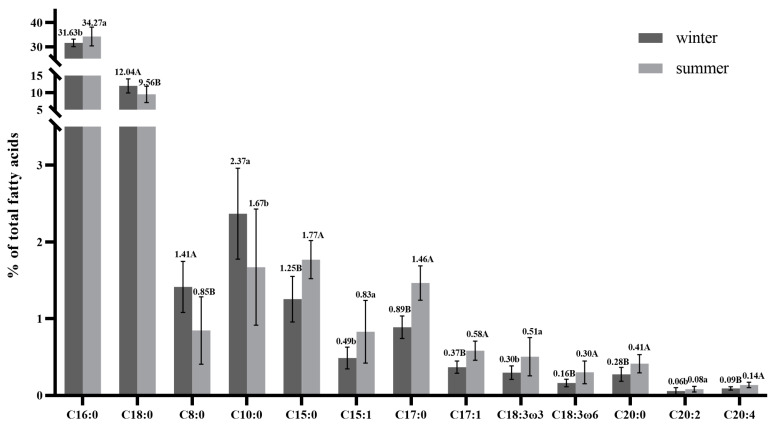
The individual fatty acids have a significant difference in winter and summer of crossbred buffalo raw milk. a, b—values differ significantly between two groups (*p* < 0.05); A, B—values differ extremely significantly between two groups (*p* < 0.01).

The concentrations of fatty acid C5:0, C7:0, C9:0, and C11:0 are not listed in Table 3, since their concentrations were less than 0.02%. As expected, the dominant fatty acids in winter and summer buffalo milk ranked in the order of C16:0, C18:1, C14:0, and C18:0, and their total amount reached up to > 75% of total fatty acids in milk, which was consistent with several reports [1,36,64]. Season was an important factor affecting the compositions of buffalo milk FA, but it had different effects on individual fatty acids. The 13 fatty acids exhibited a significant difference between winter and summer (Figure 1). Overall, the contents of C8:0, C10:0, and C18:0 were significantly higher (*p* < 0.05) in winter buffalo milk, while the contents of C15:0, C15:1, C16:0, C17:0, C17:1, C15:0, C18:3(ω-6), C18:3(ω-3), C20:0, C20:2, and C20:4 were significantly higher (*p* < 0.05) in summer milk. Similar influences of seasons on individual fatty acids have been reported in buffaloes and other ruminants from other countries [64].

**Figure 2:**
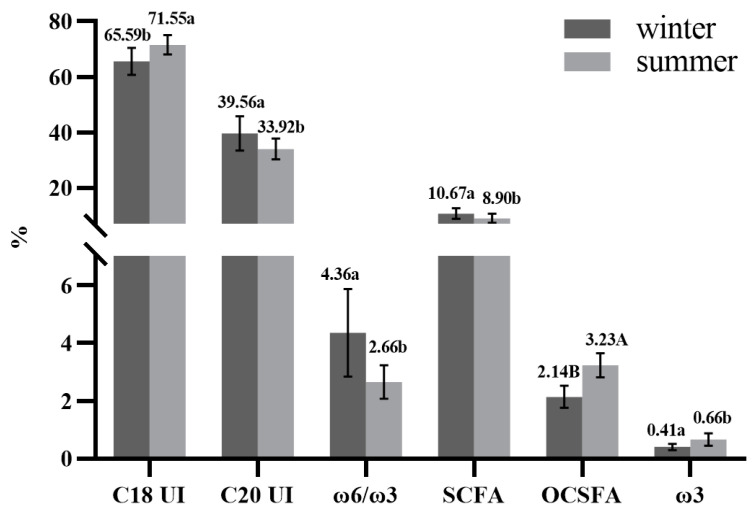
The fatty acid groups and lipid quality health indices have a significant difference in winter and summer of crossbred buffalo raw milk. a, b—values differ significantly between two groups (*p* < 0.05), A, B—values differ extremely significantly between two groups (*p* < 0.01).

Table 4 showed main fatty acid groups and lipid quality indices. Furthermore, indicators with significant difference between winter milk and summer milk have been reflected in the Figure 2. SFA exhibited the highest content in fatty acids, followed by MUFA and PUFA. The three fatty acid groups had no significant differences between the two seasons. Regarding SFA in crossbred buffalo milk, OCSFA was significantly higher (*p* < 0.01) in summer milk, and ECSFA was higher in winter milk. Winter crossbred buffalo milk contained more desirable DFA and less undesirable OFA. For PUFA, ω-3 was found to be significantly higher (*p* < 0.05) in summer milk, whereas ω-6 was lower in the winter but insignificantly. Considering the classification based of the carbon chain length, SCFA exhibited the lowest content in milk fat, with a significant difference between winter and summer crossbred buffalo milk (*p* < 0.05). MCFA and LCFA respectively displayed the highest and second highest content, with no statistical differences between the two seasons’ milk.

The values of lipid quality indices differed between winter and summer milk (Table 4). Stearoyl-CoA desaturase (SCD) is an enzyme responsible for the conversion from SFA (C14:0, C16:0, C18:0) to MUFA and CLA (C14:1, C16:1, C18:1) in the mammary gland. The higher SCD value in milk, the higher level of SFA conversion into MUFA and CLA [65]. Our data showed that all the indices of unsaturated fatty acid (C14 Index, C16 Index, C18 Index) were higher in summer milk than in winter milk. The C18 Index was significantly higher in summer milk than in winter milk (*p* < 0.05). The AI (atherogenicity index), TI (thrombogenicity index), ω-6/ω-3 ratio, and hypocholesterolemic/hypercholesterolemic (H/H) ratio are usually used to evaluated the nutrition on value of food and its health benefits. AI indicates the relationship between major FA (C12:0, C14:0, C16:0) classified as proatherogenic and antiatherogenic UFA (ω-6, ω-3, MUFA) [55]. FA are a vital mediator in the development of atherosclerosis [66]. TI demonstrates a sign of blood clot formation in the patients’ vessels, and blood clot formation is defined as the relationship between pro-thrombogenic (SFA) and anti-thrombogenic fatty acids (monounsaturated fatty acids, ω-6, and ω-3 PUFA) [55]. The high value of AI and TI represents a substantial risk of cardiovascular diseases [23,27]. Human plasma cholesterol level can be reduced by drinking milk with low AI value [67]. Our data showed that summer milk had lower AI, TI, and H/H than winter milk, but not significantly, suggesting that summer milk had superior health-promoting characteristics. Moreover, Simopoulos reported that the ω-6/ω-3 ratio is a key index reflecting the reduced risk of multiple chronic diseases, and milk of which ω-6/ω-3 ratio is below the recommended value of 4:1 is considered to be beneficial to health [68]. Summer crossbred buffalo milk exhibited a lower ratio of ω-6/ω-3 (2.66) than winter crossbred buffalo milk (4.36), indicating summer milk’s desirable quality in reducing the risk of multiple chronic diseases.

Our results showed a higher PUFA concentration in summer milk than in winter milk. The possible reason might be that in winter, relatively limited green forage feeds were available, whereas in summer, buffaloes were provided with some fresh crop by-products and residues such as wheat straw, cottonseed cake, and rice husk, in addition to forage.

### 3.3. Amino Acid Profiles

The amino acid profiles of winter and summer milk are presented in Table 5. Compared with winter buffalo milk, summer buffalo milk had a higher concentration of 17 individual amino acids, essential amino acids (EAA), non-essential amino acids (NEAA), brain-chain amino acids (BCAA), and total amino acids (TAA), which was consistent with the variation of the whole protein content (Table 2). In addition, the EAA/TAA ratio was significantly higher in summer milk than in winter milk (*p* < 0.05), and the EAA/NEAA ratio was extremely significantly higher in summer milk than in the winter milk (*p* < 0.01), which was highly consistent with previous reports on China Yak milk and donkey milk [69,70]. These results might be related to the protein proportion in herbage or vine plant feeds [71]. LEU has a vital effect on protein metabolism and the translation initiation pathway of muscle protein synthesis [72]. Our study found that LEU had the highest content among all the EAA, which was higher in summer milk than in winter milk. Although CYS accounts for a small fraction in AA, CYS was the only amino acid, with a significant difference (*p* < 0.05) between summer and winter buffalo milk. However, one recent study revealed that the content of LEU and CYS in Chinese breast milk is much lower than that recommended by the European Society for Pediatric Gastroenterology Hepatology and Nutrition [73]. Hence, buffalo milk might be a supplementary for infants in China. In the NEAA, GLU plays an important role in AA metabolism and immunity against pathogens [74]. In this study, GLU exhibited the highest content in two seasons. Compared to soy protein, milk protein contributes more to the increase in BCAA contents in peripheral tissues [75,76]. BCAA consists of ILE, LEU, and VAL, and BCAA are an important compound associated with metabolic health, especially obesity alleviation [77]. In this study, BCAA account for about 20% of total AA with a higher content in summer milk than in winter milk.

## 4. Conclusions

This study found that chemical parameters, fatty acid profiles, lipid quality indicators, and amino acid profiles differed between winter and summer crossbred buffalo milk produced in China. Summer buffalo milk exhibited a higher level of MUFA, PUFA, DFA, and ω-3 than winter buffalo milk but a lower level of major SFA. In addition, summer buffalo milk showed a higher value of unsaturated fatty acid indices (C14, C16, C18) but a lower value of relevant cardiovascular indices (IA, IT) and ω-6/ω-3 ratio. All 17 amino acids, including 8 EAA and 9 NEAA were higher in the summer buffalo milk. In conclusion, summer buffalo milk might be a desirable diet option for human health and nutrition. This study made the first attempt to compare and reveal the difference between summer and winter crossbred buffalo milk in their basic chemical parameters, fatty acid profile, and amino acid profile of buffalo in China. Our findings provide the reference for the research and development of buffalo dairy products in China or other Asian countries.

## Figures and Tables

**Table 1:** Fat, protein, polyunsaturated fatty acids, and essential amino acids of cow, goat, ewe, donkey, camel, and mare in their raw milk [5,6,7,8,9,10].

Item ^1^	Buffalo	Cow	Goat	Sheep	Donkey	Camel	Mare
Fat, g/100 g	5.3–15.0	3.5–6.4	3.0–7.2	4.0–9.0	0.3–1.8	5.0–6.2	0.4–7.2
MUFA, g/100 g of FA	24.0–29.2	2.0–30.0	19.0–36.0	23.0–36.0	14.0–30.0	26.1–34.1	18.0–36.0
PUFA, g/100 g of FA	2.3–3.9	2.4–6.3	2.6–5.6	2.6–7.3	14.0–30.0	2.7–4.42	13.0–51.0
ω3, g/100 g of FA	0.2–1.4	0.3–1.8	0.3–1.5	0.5–2.3	4.0–16.3	0.5–1.2	2.2–31.2
Protein, g/100 g	2.7–4.7	3.1–3.8	3.0–5.2	4.5–7.0	1.5–1.8	3.6–3.8	1.5–2.8
EAA, g/100 g of AA	1.7–2.0	1.8–2.0	2.1–2.7	2.5–3.0	0.6–0.7	1.7–2.0	1.0–1.5

^1^ MUFA = monounsaturated fatty acids; PUFA = polyunsaturated fatty acids; EAA = essential amino acids.

**Table 2:** Mean ± SD and range of basic parameters of crossbred buffalo raw milk in winter and summer.

Items	Winter			Summer		
	Mean ± SD	Min	Max	Mean ± SD	Min	Max
SCC (10^4^ cell/mL)	21.35 ± 18.34 a	5.30	76.60	25.51 ± 35.68 a	1.20	120.36
Fat (%)	7.25 ± 1.74 a	4.61	10.68	7.00 ± 1.54 a	4.89	9.73
Protein (%)	4.72 ± 0.60 a	3.74	5.44	5.13 ± 1.48 a	3.56	7.79
Lactose (%)	4.98 ± 0.47 a	4.12	5.52	4.30 ± 0.87 b	2.98	5.35
Total solids (%)	17.90 ± 1.82 a	14.58	20.94	17.88 ± 2.20 a	13.52	20.71

Mean = mean value; SD = standard deviation; Min = minimum value; Max = maximum value; Same letter in a row indicates no significant differences, while different letters in a row indicates a significant difference as following. a, b-values differ significantly between two groups (*p* < 0.05).

**Table 3:** Mean (g/100 g of fatty acids) ± SD and range of individual fatty acids of crossbred buffalo raw milk in winter and summer.

Items ^1^	Winter			Summer		
	Mean ± SD	Min	Max	Mean ± SD	Min	Max
C4:0	4.09 ± 0.82 a	3.01	5.41	3.86 ± 0.63 a	2.58	4.58
C6:0	2.81 ± 0.59 a	2.09	4.04	2.53 ± 0.64 a	1.41	3.80
C8:0	1.41 ± 0.33 A	0.99	2.14	0.85 ± 0.44 B	0.45	1.91
C10:0	2.37 ± 0.59 a	1.49	3.43	1.67 ± 0.76 b	0.95	3.34
C12:0	2.69 ± 0.56 a	1.77	3.53	2.16 ± 0.79 a	1.30	3.73
C14:0	11.48 ± 1.41 a	8.81	13.96	10.22 ± 2.12 a	7.23	14.24
C14:1	1.05 ± 0.26 a	0.80	1.49	1.12 ± 0.23 a	0.54	1.37
C15:0	1.25 ± 0.30 B	0.84	1.80	1.77 ± 0.25 A	1.34	2.16
C15:1	0.49 ± 0.14 b	0.32	0.73	0.83 ± 0.41 a	0.38	1.45
C16:0	31.63 ± 1.52 b	28.56	33.59	34.27 ± 3.87 a	28.50	40.06
C16:1	1.6 ± 0.56 a	1.06	2.85	1.92 ± 0.41 a	1.00	2.47
C17:0	0.89 ± 0.15 B	0.66	1.12	1.46 ± 0.22 A	1.05	1.78
C17:1	0.37 ± 0.08 B	0.23	0.49	0.58 ± 0.12 A	0.35	0.78
C18:0	12.04 ± 2.04 a	9.81	15.01	9.56 ± 2.4 b	7.16	16.33
C18:1	22.96 ± 2.94 a	18.95	30.09	23.88 ± 3.64 a	18.32	29.19
C18:2 (LA)	1.37 ± 0.50 a	0.77	2.20	1.2 ± 0.45 a	0.70	1.97
C18:3 (ALA)	0.30 ± 0.09 b	0.18	0.46	0.51 ± 0.25 a	0.26	0.98
C18:3 (GLA)	0.16 ± 0.05 B	0.08	0.27	0.30 ± 0.15 A	0.08	0.68
C20:0	0.28 ± 0.09 B	0.13	0.45	0.41 ± 0.12 A	0.25	0.61
C20:1	0.18 ± 0.05 a	0.09	0.25	0.22 ± 0.08 a	0.11	0.41
C20:2	0.06 ± 0.04 b	0.03	0.19	0.08 ± 0.04 a	0.03	0.14
C20:3	0.07 ± 0.02 a	0.04	0.09	0.09 ± 0.04 a	0.03	0.18
C20:4	0.09 ± 0.02 B	0.07	0.14	0.14 ± 0.04 A	0.07	0.20
C20:5 (EPA)	0.12 ± 0.04 a	0.06	0.18	0.16 ± 0.07 a	0.05	0.25

^1^ LA = linoleic acid; ALA = α-linolenic acid; GLA = γ-linolenic acid; EPA= eicosapentaenoic acid. Mean = mean value; SD = standard deviation; Min = minimum value; Max = maximum value; Same letter in a row indicates no significant differences, while different letters in a row indicates a significant difference as following. a, b-values differ significantly between two groups (*p* < 0.05), A, B-values differ extremely significantly between two groups (*p* < 0.01).

**Table 4:** Mean (g/100 g of fatty acids) ± SD and range of fatty acids groups and lipid quality health indices of crossbred buffalo raw milk in winter and summer.

Items ^1^	Winter			Summer		
	Mean ± SD	Min	Max	Mean ± SD	Min	Max
SFA	70.93 ± 3.30 a	63.43	76.15	68.78 ± 4.17 a	61.62	74.09
UFA	28.81 ± 3.31 a	23.66	36.39	31.02 ± 4.17 a	25.91	38.38
MUFA	26.65 ± 3.04 a	21.51	33.18	28.55 ± 3.88 a	23.22	34.71
PUFA	2.17 ± 0.55 a	1.58	3.21	2.48 ± 0.74 a	1.63	3.77
SCFA	10.67 ± 1.94 a	8.25	15.02	8.90 ± 1.71 b	6.39	12.21
MCFA	50.19 ± 3.39 a	42.80	54.28	52.51 ± 5.35 a	40.83	57.77
LCFA	38.86 ± 3.85 a	34.81	46.40	38.39 ± 5.89 a	30.02	50.32
OCSFA	2.14 ± 0.39 B	1.60	2.75	3.23 ± 0.42 A	2.44	3.94
ECSFA	68.79 ± 3.37 a	61.36	74.56	65.75 ± 4.50 a	57.68	71.36
ω3	0.41 ± 0.11 b	0.27	0.58	0.66 ± 0.22 a	0.46	1.12
ω6	1.68 ± 0.53 a	1.08	2.65	1.73 ± 0.52 a	1.04	2.59
C14 index	8.43 ± 1.93 a	5.88	11.68	9.79 ± 1.77 a	6.93	13.00
C16 index	4.78 ± 1.47 a	3.48	8.20	5.31 ± 1.10 a	3.39	7.01
C18 index	65.59 ± 4.90 b	56.44	74.42	71.55 ± 3.50 a	63.71	77.68
IA	2.84 ± 0.48 a	1.92	3.42	2.61 ± 0.67 a	1.72	3.66
IT	3.58 ± 0.51 a	2.56	4.40	3.18 ± 0.62 a	2.16	3.83
DFA	40.84 ± 3.44 a	37.00	47.53	40.58 ± 5.61 a	33.07	50.49
OFA	58.9 ± 3.44 a	52.19	62.67	59.42 ± 5.61 a	49.51	66.93
H/H	0.70 ± 0.10 a	0.59	0.91	0.71 ± 0.17 a	0.49	1.02
ω6/ω3	4.36 ± 1.52 a	2.15	6.67	2.66 ± 0.58 b	1.74	3.74
PUFA/SFA	0.03 ± 0.01 a	0.02	0.05	0.04 ± 0.01 a	0.02	0.06

^1^ SFA = saturated fatty acids (C4:0, C6:0, C8:0, C10:0, C12:0, C13:0, C14:0, C15:0, C16:0, C17:0, C18:0, C20:0); UFA = unsaturated fatty acids (C14:1, C15:1, C16:1, C17:1, C18:1, C18:2 (LA), C18:3 (ALA), C18:3 (GLA), C20:1, C20:2, C20:4, C20:5 (EPA)); MUFA = monounsaturated fatty acids (C14:1, C15:1, C16:1, C17:1, C18:1, C20:1); PUFA = polyunsaturated fatty acids (C18:2 (LA), C18:3 (ALA), C18:3 (GLA), C20:2, C20:4, C20:5 (EPA)); SCFA = short-chain fatty acids (C4:0, C6:0, C8:0, C10:0) MCFA = medium-chain fatty acids (C12:0, C13:0, C14:0, C14:1, C15:0, C15:1, C16:0, C16:1); LCFA = long-chain fatty acids (C17:0, C17:1, C18:0, C18:1, C18:2 (LA), C18:3 (ALA), C18:3 (GLA), C20:0, C20:1, C20:2, C20:4, C20:5 (EPA)); OCSFA = odd-chain saturated fatty acids (C13:0, C15:0, C17:0); ECSFA = even-chain saturated fatty acids (C4:0, C6:0, C8:0, C10:0, C12:0, C14:0, C16:0, C18:0, C20:0); DFA = hypocholesterolemic fatty acids(C14:1, C15:1, C16:1, C17:1, C18:0, C18:1, C18:2(LA), C18:3(ALA), C18:3(GLA), C20:1, C20:2, C20:4, C20:5 (EPA)); OFA = hypercholesterolemic fatty acids (C12:0, C14:0, C16:0); Mean = mean value; SD = standard deviation; Min = minimum value; Max = maximum value. Same letter in a row indicates no significant differences while different letters in a row indicate significant, a, b—values differ significantly between two groups (*p* < 0.05), A, B-values differ extremely significantly between two groups (*p* < 0.01).

**Table 5:** Mean (g/100 g of amino acid) ± SD and range of individual amino acid and amino acids groups of crossbred buffalo raw milk in winter and summer.

Items ^1^	Winter			Summer		
	Mean ± SD	Min	Max	Mean ± SD	Min	Max
HIS	0.14 ± 0.02 a	0.10	0.17	0.16 ± 0.05 a	0.11	0.27
ILE	0.23 ± 0.03 a	0.17	0.28	0.25 ± 0.08 a	0.17	0.44
LEU	0.43 ± 0.05 a	0.32	0.52	0.45 ± 0.13 a	0.30	0.78
LYS	0.36 ± 0.05 a	0.28	0.45	0.40 ± 0.13 a	0.25	0.65
MET	0.13 ± 0.01 a	0.10	0.15	0.30 ± 0.54 a	0.10	2.00
PHE	0.23 ± 0.03 a	0.17	0.28	0.25 ± 0.10 a	0.16	0.52
THR	0.21 ± 0.03 a	0.17	0.26	0.24 ± 0.08 a	0.14	0.45
VAL	0.24 ± 0.03 a	0.19	0.30	0.28 ± 0.09 a	0.19	0.51
ALA	0.14 ± 0.02 a	0.10	0.17	0.16 ± 0.06 a	0.10	0.30
ARG	0.12 ± 0.02 a	0.09	0.15	0.13 ± 0.03 a	0.01	0.21
ASP	0.34 ± 0.04 a	0.25	0.41	0.41 ± 0.12 a	0.08	0.21
GLU	0.96 ± 0.12 a	0.76	1.21	1.04 ± 0.30 a	0.68	1.71
GLY	0.08 ± 0.01 a	0.06	0.10	0.10 ± 0.03 a	0.06	0.18
TYR	0.19 ± 0.02 a	0.15	0.23	0.21 ± 0.08 a	0.13	0.43
PRO	0.37 ± 0.12 a	0.11	0.64	0.46 ± 0.12 a	0.31	0.76
SER	0.24 ± 0.03 a	0.19	0.29	0.28 ± 0.09 a	0.18	0.50
CYS	0.05 ± 0.01 b	0.04	0.06	0.07 ± 0.03 a	0.04	0.16
BCAA	0.90 ± 0.11 a	0.68	1.10	0.98 ± 0.30 a	0.66	1.73
EAA	1.97 ± 0.24 a	1.50	2.41	2.33 ± 0.85 a	1.44	4.03
NEAA	2.50 ± 0.34 a	1.91	3.06	2.85 ± 0.83 a	1.90	4.95
Total	4.47 ± 0.56 a	3.41	5.47	5.19 ± 1.61 a	3.34	8.85
EAA/NEAA	0.80 ± 0.04 B	0.71	0.90	0.81 ± 0.16 A	0.75	1.31
EAA/Total	0.44 ± 0.01 b	0.10	0.17	0.45 ± 0.04 a	0.43	0.57

^1^ LA = linoleic acid; ALA = α-linolenic acid; GLA = γ-linolenic acid; EPA= eicosapentaenoic acid; HIS = histidine; ILE = isoleucine; LEU = leucine; LYS = lysine; MET = methionine; PHE = phenylalanine; THR = threonine; VAL = valine; ALA = alanine; ARG = arginine; ASP = aspartic acid; GLU = glutamic acid; GLY = glycine; TYR = tyrosine; PRO = proline; SER = serine; CYS = cysteine. EAA = essential amino acids; NEAA = non-essential amino acids; BCAA = brain-chain amino acids. Mean = mean value; SD = standard deviation; Min = minimum value; Max = maximum value. Same letter in a row indicates no significant differences while different letters in a row indicate significant, a, b—values differ significantly between two groups (*p* < 0.05), A, B—values differ extremely significantly between two groups (*p* < 0.01).

## Data Availability

The raw data presented in this study are available on request from the corresponding author.
